# A Possible Case of Centronuclear Myopathy: A Case Report

**DOI:** 10.3390/medicina59061112

**Published:** 2023-06-08

**Authors:** Narjara Castillo-Ferrán, Juan Mario Junco-Rodriguez, Zurina Lestayo-O’Farrill, María de los Angeles Robinson-Agramonte, Zoilo Camejo-León, Héctor Jesús Gómez-Suárez, Mercedes Salinas-Olivares, Evelyn Antiguas-Valdez, Elizabeth Falcón-Lamazares, Dario Siniscalco

**Affiliations:** 1 “Comandante Manuel Fajardo” Teaching Surgical Clinical Hospital, Havana 10400, Cuba; falconelizabeth621@gmail.com; 2 Institute of Tropical Medicine “Pedro Kouri”, Havana 11400, Cuba; jmjunco83@gmail.com; 3 Institute of Neurology and Neurosurgery, Havana 10400, Cuba; zurinarabi@gmail.com (Z.L.-O.); zoilo.camejo@infomed.sld.cu (Z.C.-L.); mercedes.salina@inn.sld.cu (H.J.G.-S.); mercedes.salinas@inn.sld.cu (M.S.-O.); 4 Department of Immunochemical, International Center for Neurological Restoration, Habana 11300, Cuba; neuromary2018@gmail.com; 5 “Angel Arturo Aballí” Hospital, Havana 10400, Cuba; evelynma@infomed.sld.cu; 6 Department of Experimental Medicine, Division of Molecular Biology, Biotechnology and Histology, University of Campania, 80138 Naples, Italy

**Keywords:** muscular atrophy, muscle biopsy, hypotonia, congenital myopathy

## Abstract

Congenital myopathies (CMs) are a group of diseases that primarily affect the muscle fiber, especially the contractile apparatus and the different components that condition its normal functioning. They present as muscle weakness and hypotonia at birth or during the first year of life. Centronuclear CM is characterized by a high incidence of nuclei located centrally and internally in muscle fibers. Clinical case: a 22-year-old male patient with symptoms of muscle weakness since early childhood, with difficulty in performing physical activity according to his age, with the presence of a long face, a waddling gait, and a global decrease in muscle mass. Electromyography was performed, showing a neurogenic pattern and not the expected myopathic one, neuroconduction with reduced amplitude of the motor potential of the peroneal nerve and axonal and myelin damage of the posterior tibial nerves. The microscopic study of the studied striated muscle fragments stained with hematoxylin–eosin and Masson’s trichrome showed the presence of fibers with central nuclei, diagnosing CM. The patient meets most of the description for CM, with involvement of all striated muscles, although it is important to note the neurogenic pattern present in this case, due to the denervation of damaged muscle fibers, which contain terminal axonal segments. Neuroconduction shows the involvement of motor nerves, but with normal sensory studies, axonal polyneuropathy is unlikely, due to normal sensory potentials. Different pathological findings have been described depending on the mutated gene in this disease, but all coincide with the presence of fibers with central nuclei for diagnosis by this means, which is so important in institutions where it is not possible to carry out genetic studies, and allowing early specific treatment, according to the stage through which the patient passes.

## 1. Introduction

Congenital myopathies (CMs) are a clinically and genetically heterogeneous group of diseases that primarily affect the muscle fiber, especially the contractile apparatus and the different components that determine its normal functioning. They present as muscle weakness and hypotonia at birth or during the first year of life [1].

They have been classified according to the predominant characteristics of muscle biopsy. These include CMs with fiber disproportion, central core myopathy, minicore myopathy, nemaline myopathy, and myotubular myopathy or centronuclear myopathy (CNM), a CM sub-type characterized by skeletal muscle weakness and by increased central myonuclei [1,2].

There are many other CMs, even rare ones, such as sarcotubular and those with fingerprint-type inclusions, hyaline bodies, reducing bodies, cytoplasmic bodies, myotubular aggregates, zebra bodies or trilaminar inclusions [2].

CM is a genetic condition characterized by a high incidence of nuclei located centrally and internally in muscle fibers. Three forms have been identified:

1. Severe/lethal X-linked recessive form (myotubular myopathy) with a mutation in the MTM1 gene, which encodes myotubularin;
2. Sporadic form with variable symptoms or autosomal dominant (AD) form associated with mutation of the DNM2 gene (dynamin);
3. An autosomal recessive (AR) form related to mutations in the BIN1 (amphiphysin) gene [3,4].

The most common genes that cause CNM are MTM1, DNM2, RYR1, and TTN. Minor causative genes are BIN1, CCDC78, and SPEG [3,5]. Recently, a possible physiological expression of cannabinoid receptor (CB)-1 in skeletal muscle cells has been proposed [6]. This type of receptor, together with CB-2, belongs to the endocannabinoid system (EC), an intricate network of lipid signaling pathways, whose molecular “building blocks” are N-arachidonoylethanolamine (anandamide, AEA) and 2-arachidonoyl glycerol (2-AG) [7]. CB-1 is a G-protein-coupled cannabinoid receptor (GPCR) negatively coupled to the adenylate cyclase enzyme. In skeletal muscle, the CB-1 receptor is able to modulate the nuclear receptor (NR)-4A1 and NR4A3 mRNA gene expression; more importantly, CB-1 receptor activation leads to the pro-inflammatory interleukin (IL)-6 up-regulation in skeletal muscle [8], indicating that this type of EC receptor could be a potential pharmaceutical target for the resolution of inflammation and related disorders in skeletal muscles.

The current CM treatment is supportive. Optimal management involves a team of specialists, including neurologists, pulmonologists, physical therapists and/or rehabilitation medicine specialists, and a clinical geneticist. Tracheotomy, nasogastric tube feeding, and assisted communication equipment are frequently required. Ophthalmologists, orthopedists, and dentists must treat specific medical complications [9].

As of late 2019, an investigational product, DYN101, is in clinical development and approval for the treatment of CM patients with mutations in the MTM1 or DYN2 genes. DYN101 is an antisense oligonucleotide that is expected to decrease the production of a protein, called dynamin 2, which is found in high concentrations in patients with X-linked CNM and is thought to be overly active in patients with mutations in the DYN2 protein [10]. The increasing identification of new genes and phenotypes associated with already known genes was largely possible thanks to the advancement of next-generation sequencing techniques. While these results offer the undoubted advantage of a better diagnosis, as a limitation, they are accessible only in a few institutions. However, this case report describes the possibility to obtain a correct CM diagnosis when it is not possible to perform the genetic test. Knowing better the phenotypic spectrum of these entities, as well as their anatomopathological description, allows us to establish a phenotype/pathological correlation in some sub-groups. A better understanding of the pathophysiology and natural history of these diseases is essential for the development of new therapies [1].

## 2. Clinical Case

We present here the case of a 22-year-old male patient who is white, right-handed, and of urban origin. He shows a personal medical history of bronchial asthma since childhood, including mitral valve prolapse and allergic rhinitis. Product of a dystocic delivery by cesarean section, mother with group 0 and Rh(−) factor, 4th delivery.

His psychomotor development was apparently normal in his early years, as he was able to sit without needing support at 6 months of age, and walked without support at 12 months, although his mother noted that he did not run as fast as other children his age. During school life, he could not perform physical exercises such as bars, squats, planks, or sit-ups. He began to walk on his toes, for which he underwent surgery, with a diagnosis of shortened Achilles tendon, which improved these symptoms. Currently, it is difficult for him to go up and down stairs, or get up from the floor or a chair. He also presents frequent falls and easy exhaustion associated with a generalized decrease in muscle mass.

**Figure 1:**
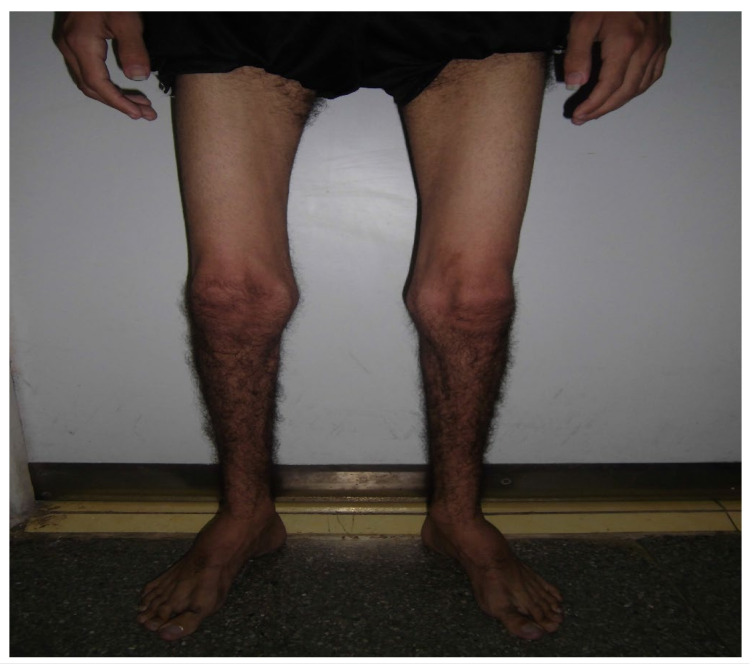
Overall decrease in muscle mass.

Physical exams:

- Posture: slight right convex scoliosis. Hyperlordosis. Increased base of support;
- Gait: waddling. Weight: 43 kg. Size: 1.71 cm. BMI: 14.72;
- Osteomyoarticular system: both high-arched feet;
- Oral cavity: arch of the high palate;
- Muscle tone: decreased globally (Figure 1).

**Figure 2:**
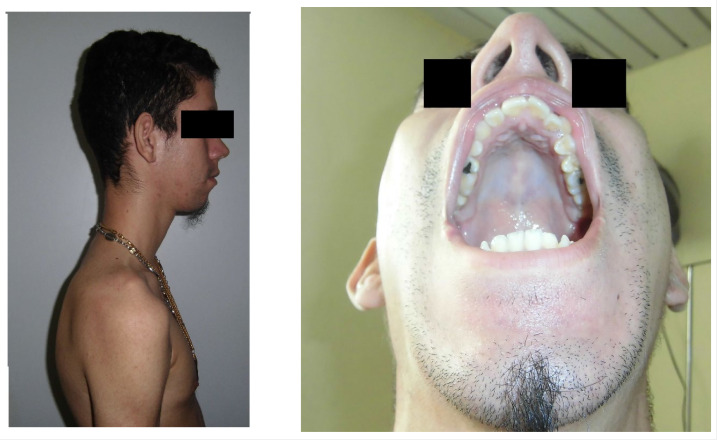
Facial deformities, such as an elongated face, micrognathia, or high-arched palate.

Facie: Facial deformities (elongated, narrow, bilateral, and symmetrical palpebral ptosis, micrognathia, high-arched palate) (Figure 2).

Trophism: global decrease in muscle mass.

Muscular strength: decreased in the 4 limbs: 3/5, proximally and distally.

Osteotendinous reflexes: globally abolished.

- Clinical laboratory: normal hematology and blood chemistry. Creatinkinase: 100 UI/L.
- Imaging:
- Abdominal ultrasound: normal.
- Chest X-ray: normal.
- Neurophysiological studies:

### 2.1. Needle Electromyography

Presence of scant spontaneous activity of the positive sharp wave (PSW) type, isolated fibrillations and fasciculations, high-frequency lesional and pseudomyotonic discharges. Chronic neurogenic pattern of mild to moderate intensity, where there are signs of denervation and re-innervation, there is no evidence of myopathic injury by EMG.

### 2.2. Neuroconduction

Sensory nerve conduction: normal median, sural and posterior tibial nerve.

Motor nerve conduction:

1. Right peroneal nerve: Decrease in the amplitudes of the combined motor potential of the muscle in the distal and proximal segments. The reduction in motor potential amplitude could be attributed to signs of muscle hypotrophy in the recording muscles;
2. Right posterior tibial nerve: Axonal damage in distal and proximal motor fibers;
3. Left posterior tibial nerve: Myelin damage in proximal motor fibers.

Wave F: upper and lower limbs in ulnar and tibial nerves, normal.

**Figure 3:**
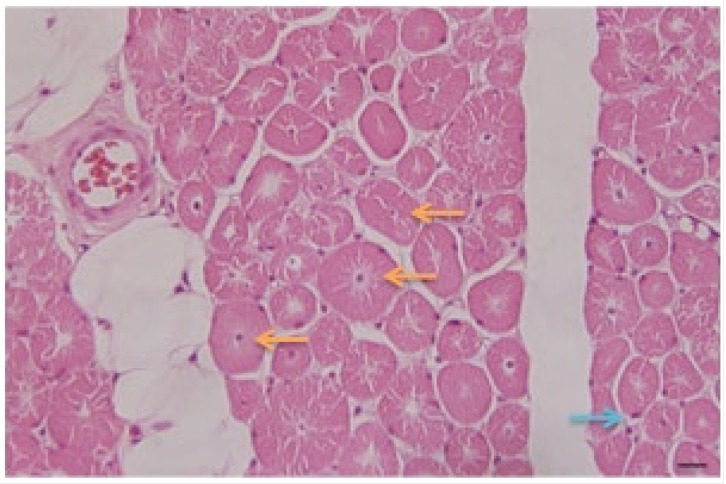
Microscopic study of skeletal muscle fragments with hematoxylin–eosin staining. Increased number of internalized nuclei in the center of the muscle fiber is observed. Surrounding most of the centrally placed nuclei there is a clear zone, with a lack of organization of contractile elements. Central nuclei are indicated by orange arrows; some normal (peripheral) myonuclei are also displayed by blue arrows. Scale bar: 50 μm.

- Neurogenetics. DNA study: Molecular study for spinal muscular atrophy, with PCR-RFLP technique. Direct detection. Result: No exon 7 gene SMN1/SMN2 deletion.
- Pathology: Biopsy of left deltoid striated muscle fragment was performed. The microscopic study of the studied fragments stained with hematoxylin–eosin (H/E) (Figure 3) and Masson’s trichrome showed:

1. Presence of fibers with central nuclei;
2. Presence of atrophic fibers;
3. Fibers of normal diameters and some hypertrophic ones;
4. Presence of clear perinuclear spaces and others with multiple central tunnels as an expression of myofilament loss;
5. No inflammation or macrophage phenomena were observed;
6. No increase in connective tissue is appreciated;
7. Discrete fatty infiltration in the perimysial center.

Diagnosis: centronuclear congenital myopathy.

## 3. Discussion

CMs are relatively non-progressive or very slowly progressing. Muscle fibers are not lost, but within each fiber, a characteristic morphologic abnormality arises [11].

In the case of this patient, there was no neonatal or infancy onset pattern, with the first symptoms appearing in early childhood. Since the disease was initially mild, there is no way to determine if it was found since the patient was born. The patient meets most of the description for CM, with involvement of all striated muscles, thin and flexible extremities, palpebral ptosis, paralysis of ocular muscles, weakness of facial, masticatory, lingual, pharyngeal, laryngeal, and cervical muscles, which is more frequently the complete picture in children, less so in adulthood. Taking into account the classification based on its severity and mode of presentation, one could think of a less severe autosomal recessive type of late infancy or childhood [11].

In the electromyographic study, both positive sharp waves and fibrillation potentials are action potentials of individual muscle fibers. These pathological forms of spontaneous activity are due to the formation of new voltage-gated sodium and calcium channels in the membranes of denervated muscle fibers, which makes the muscle fibers capable of spontaneous activation. They appear when there is denervation of muscle fibers, and there is uncoupling of the muscle fiber from its alpha motor neuron. The cause that gives rise to spontaneous activity is the denervation of muscle fibers as a result of injury to terminal axonal segments, contained within the diseased muscle tissue.

High-frequency discharges, or pseudomyotonic discharges, are repeated discharges of action potentials from groups of muscle fibers that begin and end abruptly, and are seen in neurogenic and myopathic processes.

In motor unit potentials, the duration is related to the density and diameter of the muscle fibers in the motor unit. It will increase in chronic neurogenic processes and chronic re-innervation processes and will decrease in acute-onset myopathies, due to a decrease in the number and size of diseased muscle fibers. In a chronic myopathic process, when healthy fibers remain, these fibers hypertrophy to meet the demand for muscle contraction, and then the motor unit potential increases in duration.

Observing the muscle involvement of the patient and the expected myopathic pattern, the mild to moderate chronic neurogenic pattern seen here is striking. In this study, the chronic neurogenic pattern of a mild to moderate intensity that is observed in the electromyography is striking: there are signs of denervation and re-innervation, with no evidence of myopathic injury.

A chronic myopathy with denervating features is one of the most difficult patterns to recognize. After denervation, some re-innervation can normally occur. As the condition becomes chronic, this leads to complex myopathic and neuropathic patterns, often in the same muscle. However, the degree of neuropathic changes (long, large, and polyphasic) frequently appear highly abnormal because of a slight reduction of recruitment patterns, an important clue to a possible chronic myopathy.

In the neuroconduction analysis, despite being in the presence of myopathic involvement, axonal and myelin damage was observed in the right and left posterior tibial nerve, respectively.

During routine studies, motor nerve conduction is performed before sensory nerve conduction. If a diffuse pattern of low motor amplitudes emerges, along with normal latencies, and delayed responses, axonal loss and polyneuropathy would be thought of first. If sensory studies are subsequently normal, and there is an unusual abnormal pattern of pure motor nerve conduction, axonal polyneuropathy is unlikely, given normal sensory potentials.

This pattern can occur in the following conditions: motor neuron disease, radiculopathy/polyradiculopathy, neuromuscular junction disorder (especially presynaptic), and myopathy, as seen in this patient [12].

Different pathological findings are described in the literature depending on the mutated gene in this disease. Muscle biopsy in young males presenting with the X-linked form of MTM1 shows the presence of centrally located nuclei and an evident subsarcodermal pale halo with oxidative enzymes.

In mutations in the amphiphysin 2 (BIN1) gene, aggregates of centrally located nuclei and homogeneous populations of rounded type 1 atrophic fibers can be found.

Increased connective tissue and fibro-adipose replacement without necrosis and regeneration can be observed.

In the mutation in the dynamin-2 (DNM2) gene, the muscle biopsy shows a high percentage of localized nuclear internalization, especially in small hypotrophic fibers. Numerous fibers show the “sarcoplasmic radiating strands” [13,14].

In this patient, no genetic studies were performed to determine the affected gene and the diagnosis was made based on the clinical picture and the neurophysiological and pathological studies. However, several pathological features are described that are present in some of the mutations and inheritance patterns. It would be difficult to determine the genetic diagnosis from the description observed under the microscope. However, the general pattern of this description allows it to be defined as a centronuclear myopathy. Indeed, through stretched muscle biopsy it is possible to determine the diagnosis of centronuclear myopathy since the histopathological description is pathognomonic. The outstanding pathologic features of the disease are the smallness of muscles and their constituent fibers and central nucleation. Muscle biopsies reveal myonuclei in the center of muscle fibers, often forming chains when viewed longitudinally. Surrounding most of the centrally placed nuclei there is a clear zone, in which there is a lack of organization of contractile elements, a description that matches the case (Figure 3). Other investigations have followed this approach in studies of this pathology [15]. Agrawal et al. suggest that between 60–80% of diagnoses of myopathies are made genetically [16]. A recent Danish study showed that only 56% of 107 cases of congenital myopathies older than 5 years old received a genetic diagnosis. The rate of diagnosis was in 83% of the cases with a muscle biopsy of specific characteristics and 29% with non-specific histology [15,17].

Although congenital myopathies have a wide clinical spectrum, the fact of objectifying the changes in the evolution of any patient should be a pillar in any diagnostic process. This makes it possible to establish an assertive prognosis from the functional point of view, establish treatment goals, and appropriately discern the different therapeutic options at a given time, depending on the course of the disease.

## 4. Conclusions

A clinical case of a 22-year-old male patient with symptoms of CM is presented here. The patient showed muscle weakness since early childhood, with difficulty in performing physical activity according to his age, with the presence of a long face, a waddling gait, and a global decrease in muscle mass. Electromyography and microscopic studies diagnosed CM. The patient meets most of the description for CM, with involvement of all striated muscles, although it is important to note the neurogenic pattern present in this case, due to the denervation of damaged muscle fibers, which contain terminal axonal segments. For this patient, no genetic analysis was performed to determine the affected gene; the diagnosis was made based on clinical imaging and neurophysiological and pathological analysis. The presence of fibers with central nuclei was a key point for the CM diagnosis. This case report shows the possibility of correctly diagnosing the CM without a genetic test when it is not possible to perform.

## Data Availability

Data is unavailable due to the privacy of the patient.

## References

[1] De Benito D.N., Ortez C., Carrera Garcia L., Expósito J., Bobadilla E., Nascimento A. (2019). Diagnotico y tratamiento de las miopatías congénitas. Medicina.

[2] Zarranz Imirizaldu J.J. (2018). Neurologia.

[3] Rodríguez-Zambrano L., Orjuela-Rolón C., Ortiz-Corredor F., Espinosa García E. (2017). Evaluación funcional en paciente con miopatía congénita centronuclear asociada a Diamina 2. Fisitria.

[4] Ortega X., Corral G., Rojas G., Carrizo J., Suárez B., Castiglioni C. (2018). Magnetic resonance of complete body for muscle study and quantification of fatty fraction in pediatric patients with congenital myophaties. Rev. Médica Clínica Condes.

[5] Pelin K., Wallgren-Pettersson C. (2019). Update on the Genetics of Congenital Myopathies. Semin. Pediatr. Neurol..

[6] Haddad M. (2021). The Impact of CB1 Receptor on Nuclear Receptors in Skeletal Muscle Cells. Pathophysiology.

[7] Brigida A.L., Schultz S., Cascone M., Antonucci N., Siniscalco D. (2017). Endocannabinod Signal Dysregulation in Autism Spectrum Disorders: A Correlation Link between Inflammatory State and Neuro-Immune Alterations. Int. J. Mol. Sci..

[8] Haddad M. (2021). The Impact of CB1 Receptor on Inflammation in Skeletal Muscle Cells. J. Inflamm. Res..

[9] Dowling J.J., Lawlor M.W., Das S., Adam M.P., Everman D.B., Mirzaa G.M., Pagon R.A., Wallace S.E., Bean L.J.H., Gripp K.W., Amemiya A. (1993). X-Linked Myotubular Myopathy.

[10] Dynacure (2020). https://www.dynacure.com/pipeline/.

[11] Ropper A.H., Samuels M.A., Klein J.P., Prasad S. (2019). Adams and Victor’s Principles of Neurology.

[12] Preston D.C., Shapiro B.E. (2020). Electromyography and Neuromuscular Disorders.

[13] Malfatti E. (2018). Miopatías congénitas. Rev. Médica Clínica Condes.

[14] Kumar V., Abbas A.K., Aster J.C. (2015). Robbins & Cotran Pathologic Basis of Disease.

[15] Quintana-Vega V., Barragán-Pérez E.J., Alarcón-De la Luz E., Alarcón-Cabrera E., Sadowinski-Pine S., Aguirre-Hernández J. (2022). Fenotipo de miopatía congénita central core autosómica dominante con alteraciones del gen RYR1. A propósito de un caso clínico. Acta Pediatr. Mex..

[16] Agrawal P.B., Pierson C.R., Joshi M., Liu X., Ravenscroft G., Moghadaszadeh B., Talabere T., Viola M., Swanson L.C., Haliloğlu G. (2014). SPEG interacts with myotubularin, and its deficiency causes centronuclear myopathy with dilated cardiomyopathy. Am. J. Hum. Genet..

[17] Witting N., Werlauff U., Duno M., Vissing J. (2017). Phenotypes, genotypes, and prevalence of congenital myopathies older than 5 years in Denmark. Neurol. Genet..

